# NanoForms: an integrated server for processing, analysis and assembly of raw sequencing data of microbial genomes, from Oxford Nanopore technology

**DOI:** 10.7717/peerj.13056

**Published:** 2022-03-29

**Authors:** Anna Czmil, Michal Wronski, Sylwester Czmil, Marta Sochacka-Pietal, Michal Cmil, Jan Gawor, Tomasz Wołkowicz, Dariusz Plewczynski, Dominik Strzalka, Michal Pietal

**Affiliations:** 1 Department of Complex Systems, Rzeszow University of Technology, Rzeszow, Subcarpathian, Poland; 2 Department of Biotechnology and Bioinformatics, Rzeszow University of Technology, Rzeszow, Subcarpathian, Poland; 3 DNA Sequencing and Oligonucleotide Synthesis Laboratory, Institute of Biochemistry and Biophysics, Polish Academy of Sciences, Warsaw, Masovian, Poland; 4 Department of Bacteriology and Biocontamination Control, National Institute of Public Health-National Institute of Hygiene, Warsaw, Masovian, Poland; 5 Laboratory of Functional and Structural Genomics, Centre of New Technologies, University of Warsaw, Warsaw, Masovian, Poland; 6 Laboratory of Bioinformatics and Computational Genomics, Warsaw University of Technology, Warsaw, Masovian, Poland

**Keywords:** NGS, Bioinformatics, Oxford Nanopore, Genomics, Webserver, DNA sequencing, DNA assembly, Microbial genomes

## Abstract

**Background:**

Next Generation Sequencing (NGS) techniques dominate today’s landscape of genetics and genomics research. Though Illumina still dominates worldwide sequencing, Oxford Nanopore is one of the leading technologies currently being used by biologists, medics and geneticists across various applications. Oxford Nanopore is automated and relatively simple for conducting experiments, but generates gigabytes of raw data, to be processed by often ambiguous set of alternative bioinformatics command-line tools, and genomics frameworks which require a knowledge of bioinformatics to run.

**Results:**

We established an inter-collegiate collaboration across experimentalists and bioinformaticians in order to provide a novel bioinformatics tool, free for academics. This tool allows people without extensive bioinformatics knowledge to simply process their raw genome sequencing data. Currently, due to ICT resources’ maintenance reasons, our server is only capable of handling small genomes (up to 15 Mb). In this paper, we introduce our tool, NanoForms: an intuitive and integrated web server for the processing and analysis of raw prokaryotic genome data, coming from Oxford Nanopore. NanoForms is freely available for academics at the following locations: http://nanoforms.tech (webserver) and https://github.com/czmilanna/nanoforms (GitHub source repository).

## Introduction

Next Generation Sequencing technologies, such as Illumina (Gloor et al., 2010), Pacific BioSciences (Rhoads & Au, 2015) and Oxford Nanopore (Jain et al., 2016), dominate today’s landscape of genetics and genomics research. Each technology has its advantages, disadvantages and market share in specific applications and niches. The applications of Oxford Nanopore technology include eDNA extraction and sequencing (*i.e.,* Garlapati et al., 2019), rapid viral sequencing (including the current challenge of SARS-CoV2 (Wang et al., 2020)), human genome comparative sequencing (De Coster et al., 2019) and many others. Short-read sequencing technologies such as Illumina have made bacterial genome sequencing relatively cheap and accessible. However, the procedure of closing microbial genomes is often costly and laborious. Assembly of short reads from genomes that are repetitive and/or have extreme %GC content remains challenging. These difficulties can be mostly overcome by using single-molecule, long-read sequencing technologies such as the Oxford Nanopore. Nanopore helps with closing bacterial genomes (Risse et al., 2015; Kawalek et al., 2020), can deliver two strategies for bacterial genome assembly (Goldstein et al., 2019), even helps to obtain complete bacterial chromosomes from microbiomes (Moss, Maghini & Bhatt, 2020) or is used in routine microbial genome sequencing (Wick et al., 2017a). Two main strategies are used to assemble bacterial genomes using long read sequencing. In the first, nanopore reads are used for long read only genome assembly followed by polishing with Illumina reads. Alternatively, long reads are used to enhance genome assemblies that are generated from short-read Illumina data. In such case nanopore reads can scaffold contigs generated by short read sequencing. It is also now possible to extract 3D structures of the genome using Oxford Nanopore (Ulahannan et al., 2019).

Oxford Nanopore is relatively user-friendly, easily operated, inexpensive, and can be simply adjusted to allow for rapid sample processing (including outdoor usage). The downside is, after the experiment is done, researchers are left with a huge amount of raw data. There is a wide variety of software tools available to perform taxonomic classification of the raw data. There are also many comparative studies that evaluate the top performing bioinformatics tools, provide recommendations for use cases, and show how to run these tools (McIntyre et al., 2017; Escobar-Zepeda et al., 2018; Simon et al., 2019). An inexperienced user, however, could easily become overwhelmed by the complexity of the data, the fast pace of tool development, and the version updates, command changes, installation problems, etc.

## Materials & Methods

**Figure 1:**
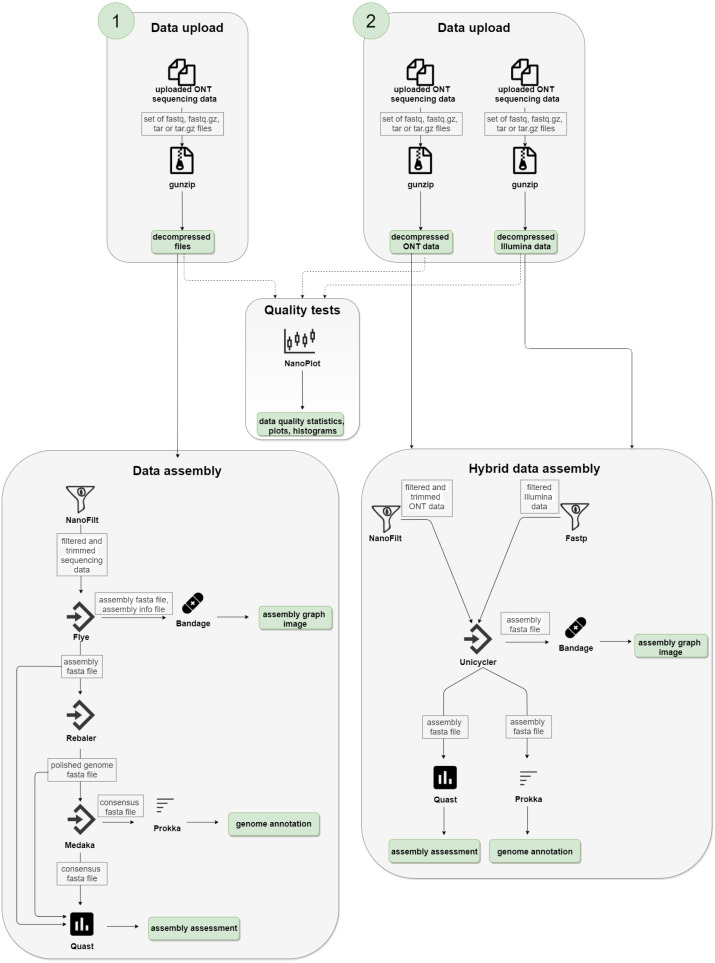
NanoForms server workflow. Subsequent computation steps are run but in between, the user is given the partial results can take action, *i.e.*, give more specific parameters for the next programmes, based on the data available (*i.e.*, quality, quantity *etc*.). In the end, the report is generated and sent in a form of a PDF file.

We used the following technologies to create the NanoForms server: Python language, Linux/UNIX/BSD operating system, Django application server, Workflow Description Language and Cromwell, Crontab, Docker and BioContainers (Da Veiga Leprevost et al., 2017), and a custom set of bioinformatics tools. The NanoForms server is freely available for academic use and a commercial release of the server (for non-academics, businesses, etc.) is planned. We also provide its source code (under GPLv3 license) for non-commercial uses. The server is fully virtualized, with about 30 processor cores and 120 GB RAM available on average. The infrastructure is also hosted in a virtual environment. It can handle about 5-10 parallel jobs (taking into account dataset size limits of 15 GB). In general, single run processing time depends on configuration, sample size and the assembly type (short reads, long reads or both). It takes nearly two hours to process a 220 MB sample of ONT data, yielding the results. Computation time grows in a near linear manner, with a 1 GB dataset taking about five hours. Hybrid assemblies, which include a Unicycler polishing stage, can take up to 10 h for a 1 GB dataset combined (300 MB of ONT and 600 MB of Illumina data). However, actual run times will depend on server usage, so for complete control over timings one can install a local version of nanoforms. The detailed diagram of the server workflow is shown in Fig. 1.

The current version of the server includes the use of the following bioinformatics applications: Nanoplot 1.32.0 NanoFilt 2.7.1 (De Coster et al., 2018), FastQC 0.11.9 (https://www.bioinformatics.babraham.ac.uk/projects/fastqc/), Flye 2.8.1 (Lin et al., 2016), Bandage 0.8.1 (Wick et al., 2015), Rebaler 0.2.0 (https://github.com/rrwick/Rebaler), Medaka 1.0.1 (https://github.com/nanoporetech/medaka), Quast 3.2  (Mikheenko et al., 2018), Fitlong 0.1.0 (https://github.com/rrwick/Filtlong), Prokka 1.14.6 (Seemann, 2014), Kraken Tools 0.1 (Davis et al., 2013), Kraken 2.1.0 (Wood, Lu & Langmead, 2019) and Krona 2.7.1 (Ondov, Bergman & Phillippy, 2011). For hybrid genome assembly, we use Unicycler 0.3.0b (Wick et al., 2017b) and Fastp 0.18.0 (Chen et al., 2018) for filtering data from Illumina. A hybrid assembly strategy has been developed to overcome the limitations of both Illumina and Oxford Nanopore sequencing and to unlock their full potential for genome assembly. Oxford Nanopore long reads can scaffold contigs generated by Illumina short reads to disambiguate regions of the assembly graph that cannot be resolved by Illumina short reads alone, as implemented in the Unicycler assembler (Chen, Erickson & Meng, 2020).

The human genome comprises approximately 3 Gb of nucleotides while a typical raw data set from Oxford Nanopore sequencing (before base-calling) exceeds 1 TB. This makes it difficult to upload the data with even high-speed bandwidth (a 100 Mbps transfer would take over 24 h to transfer the data alone). In addition, such large amounts of data would require substantial funding for computing resources as the required calculations would be considered Big Data. Because of these technological issues, we narrowed the analyses to genomes of prokaryotic sizes (up to 15 Mb in length, up to 15 GB in file size) but our server also can handle small eukaryotic genomes (such as S. cerevisiae). Unicycler may not be the best tool for yeast genomes but Flye and nanopore assembly pipeline from NanoForms, might be easily used for small euakryotic genomes (see: Martín-Hernández et al., 2021). After deploying this server and gathering remarks from the users, we are considering designing another milestone which might address this problem. We also plan to launch the commercial version of the server for institutional clients.

## Results

We introduce NanoForms: an intuitive and integrated web server for the processing and analysis of raw data from small genomes, yielding from Oxford Nanopore technology. The user uploads an archived, single sequence file (FASTQ) or a list of archived, sequence files, then the data is preprocessed. The user then chooses several options on the go and, after subsequent steps, the user obtains the DNA/RNA sequence in a form of FASTA file as well as the HTML summary with reports, images or statistics of the calculations performed.

**Figure 2:**
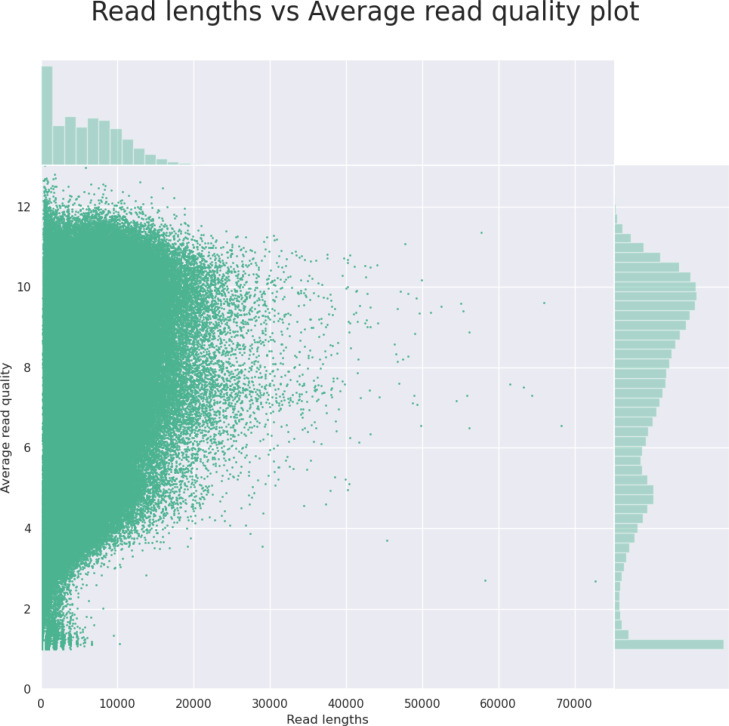
An example histogram for read length *vs.* the read quality, as the output from the NanoPlot tool. Based on this information on the server website, the user can decide if the quality of the data is good enough to continue the analysis and thus, save time of the project or decide to which extent to crop the data to exclude low-quality short reads.

**Figure 3:**
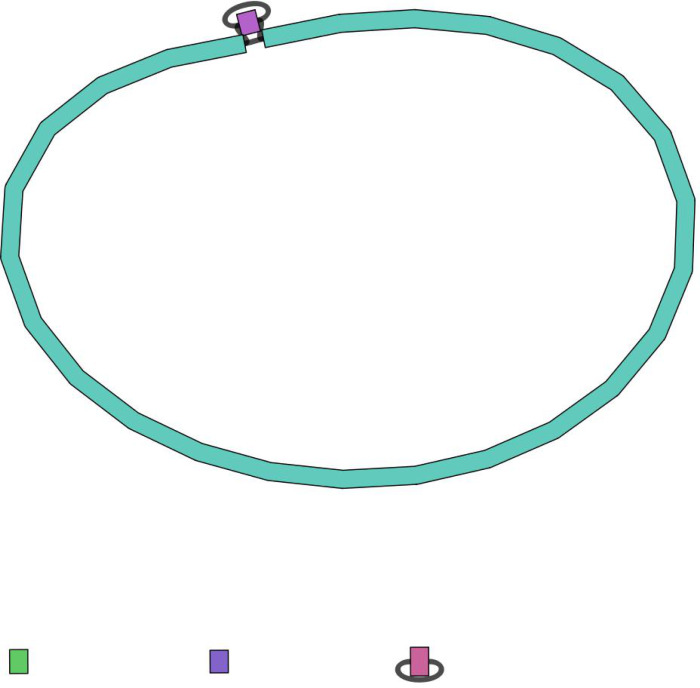
An example of a circular sequence, being assembled at the end, as visualized by the Bandage tool. Sometimes the long-read nanopore data gives a few separate genome fragments as sequencing output (contigs), so the hybrid assembly option, provided by NanoForms, can often resolve these ambiguities. This image is normally the last stage of NanoForms sequencing protocol, however all figures and statistics that arose on the course of sequencing, are delivered to the user as a report.

The initial output from the NanoPlot program (read length *vs.* the read quality) gives the user a quick outlook (as shown in Fig. 2) that helps him or her decide whether to continue the analysis or to go back to the lab to fix the sample. The final output of the NanoForms service is an assembled genome in fasta format, prokka annotation files and Bandage diagram, allowing for easy graphical assessment of assembly completeness. An example, derived for the Bacillus subtilis, is depicted in Fig. 3. Since the MinION is marketed as needing only minor laboratory skills to operate, NanoForms needs practically no bioinformatics skills to produce the sequence (however more skilled users can benefit from extra, but optional, commands and options that they can provide during the course of the analysis). Therefore, we claim that our NanoForms server, in combination with Oxford Nanopore technology, has ultimately made NGS available for all, including both biologists and bioinformaticians, as specialized skills are no longer needed to perform certain NGS tasks and analyses. On the server website, after logging in, there are several toy datasets already provided to the user. Users can use these datasets for quality tests or data assembly using the respective forms provided in NanoForms, such as Bacillus subtilis SRX6978160.

## Discussion

We performed a detailed analysis and comparison of similar services available for researchers. The first service to be tested was the CGE  (Larsen et al., 2017) server. We checked and tested this service at the very beginning of this project and at that time, it was an up-to-date and convenient service, and easy to use for biologists without technical knowledge. Shortly before drafting this manuscript, the part of the server dedicated to genome assembly went offline, so the only input is the contigs in FASTA (Pearson, 1990) format. The rest of the tools tested are free to use, but as standalone programs, they are not interconnected with the comprehensive pipeline. In addition, no figures are generated, which makes the qualitative analyses more difficult.

The Enterobase server (Zhou et al., 2020) is aimed at wgMLST analyses. This server is mainly designed for genotyping isolates. Users can screen the database against specific STRs etc., and the service can also generate phylogenetic trees. Enterobase is dedicated to analyses of gut bacteria and supports Illumina or PacBio reads. The user only needs to provide the FASTQ (Cock et al., 2010) files, which need to be compressed by the gzip tool and also, manually curated, before running the service. The figures can be generated or plotted, however this requires additional manual user intervention. The Enterobase server does not accept nanopore data.

Another interesting tool, though with significantly diminished accessibility, is the Galaxy Tools service (Cock et al., 2013). Our experience testing this service suggests that the stand-alone version of the server needs to be installed and run locally for optimal use, but for smaller analysis it can be also run on the public server. The server provides workflows (though only an empty set, for the novel user) which the user can adjust upon request. The tool supports both Illumina and long read (nanopore, PacBio) data input. It is worth noting that both the CGE and Galaxy Tools offer additional applications for practical genome analysis such as: resistance, serotype or virulence, and maintains the updated databases of these genetic targets.

**Table 1:** NanoForms server *vs.* other services: the comparison. Our service successfully fills in the gap for NGS genome assembly, in regard to the fully automated (but interactive) pipelined nanopore data processing.

**Feature**	**CGE**	**Enterobase**	**Galaxy tools**	**EPI2ME**	**NanoPipe**	**Patric**	**Nano galaxy**	**NanoSPC**	**NanoForms**
Raw data processing	+	+	+	+	+	+	+	+	+
Interactive interface	+	+	+	+	+	+	+	+	+
QA, reports	+	+	+	+	+	+	+	+	+
Free for academics	+	+	+	+	+	+	+	+	+
Sequence assembly	−	+	+	−	+^a^	+	+	+	+
Qualitative analyses: images	−	+	+	+	+	+	+	+	+
Nanopore data processing	+	−	+	+	+	+	+	+	+
Tools connected into the pipeline	−	+	+/ −^b^	−	**-**	+/ −^b^	+	+	+
Hybrid assembly: nanopore + Illumina	−	−	+	−	**-**	+	+	**-**	+
Special nanopore visualization tools	−	−	+	+	**-**	−	+	+	+
Summary report generated	−	−	−	−	+	−	−	+	+
Ease of use^c^	+	+/ −	+/ −	+	+	+	+	+	+
Dry-lab knowledge unnecessary^c^	+	+	−	−	+	−	−	+	+

**Notes.**

a But not the novo assembly.

b There are no automatic pipelines, but users can develop them themselves.

c Subjective assessments of the authors.

While preparing and programming our tool, a software report was published about the new toolkit, NanoGalaxy, dedicated to the nanopore data processing (De Koning et al., 2020). NanoGalaxy is an extension of the aforementioned Galaxy Tools, in the area of support for nanopore data. In some ways NanoForms and NanoGalaxy seems to have similar features and functionalities. Similar to standard Galaxy Tools, NanoGalaxy seems to be more powerful but on the other hand it is aimed at more advanced user with more bioinformatics skills. In our subjective opinion NanoForms is easier to use for non-bioinformatics users but on the other hand can be treated as a quite bordered “black box”. NanoGalaxy is more complex and has more functionalities, but getting familiar with the numerous available algorithms requires some bioinformatics experience. NanoGalaxy and NanoSPC (Xu et al., 2020) deliver similar results and has similar capabilities as our server. NanoSPC is focused only on Nanopore data and as a result it is not possible to perform *e.g.*, hybrid assembly there. It is also focused mostly on metagenomics, identification of pathogens and variant calling, but it seems to be easy to use also for users with limited dry-lab knowledge.

Another tool we tested was Patric (Gillespie et al., 2011). This platform provides bioinformatics analyses of all bacteria, with special focus on pathogens. It supports hybrid genome assembly in the formula of short + long reads (PacBio and nanopore are supported). Table 1 provides the comprehensive summary of the features about the quoted services, compared based on NanoForms functionality, in terms of nanopore data processing. We decided not to include “demultiplexing reads support” in our table as the most current version of MinKnow software (the native software to Oxford Nanopore) supports this feature already.

As we were pursuing this project in mid-2020, Oxford Nanopore announced the availability of their EPI2ME (http://epi2me.nanoporetech.com/,requireslogin) cloud-based workflow to process raw nanopore data. The platform’s intended use is only nanopore data analysis, without options for sequence assembly or trimming. After performing the base calling, reads are uploaded to the server *via* the EPI2ME Agent, which is a stand-alone program. The user can pick one of the several workflows (ie. microbiological classification or human genome analysis) which are triggered and executed in real time. Also in the table, the reader might find the characteristics of the last tool we reviewed: NanoPipe (Shabardina et al., 2019).

## Conclusions

In summary, our NanoForms server, freely available for all academics, bridges the high-speed of prokaryotic genome assembly with an intuitive, interactive interface. According to the Oxford Nanopore MinION specification product page, it is sufficient for the user to have a mid-range laptop and the device to obtain the sequence of the sample. No further resources are needed and the user can continue the genomic analyses after a short break in sequencing with the use of NanoForms service.

## Additional Information and Declarations

### Data Availability

The following information was supplied regarding data availability: The source code of the NanoForms server is available at Github: https://github.com/czmilanna/nanoforms. This is the source for standalone server installation. The server is available at https://nanoforms.tech/. The Bacillus subtilis sequences are available at ENA: SRX6978160.
